# Students and physicians differ in perception of quality of life in patients with tumors of the upper gastrointestinal tract

**DOI:** 10.1038/s41598-024-59350-7

**Published:** 2024-04-24

**Authors:** Lena Schooren, Grace Oberhoff, Sandra Schipper, Alexander Koch, Andreas Kroh, Steven Olde Damink, Tom F. Ulmer, Ulf P. Neumann, Patrick H. Alizai, Sophia M. Schmitz

**Affiliations:** 1 Department of General, Visceral- and Transplantation Surgery, RWTH Aachen University Hospital, Pauwelsstr. 30, 52074 Aachen, Germany; 2 Department of Gastroenterology, Digestive Diseases and Intensive Care Medicine, RWTH Aachen University Hospital, Pauwelsstr. 30, 52074 Aachen, Germany; 3 Department of Surgery, Maastricht University Medical Center, P. Debyelaan 25, 6229 HX Maastricht, The Netherlands; 4 Department of Trauma and Reconstructive Sugery, BG Klinikum Bergmanntrost, 06112 Halle, Germany; 5 Department of General-, Visceral- and Transplantation Surgery, University Hospital Essen, Hufelandstr. 55, 45147 Essen, Germany; 6 Klinik für Allgemein- und Viszeralchirurgie, Gemeinschaftskrankenhaus Bonn, Prinz-Albert-Straße 40, 53113 Bonn, Germany

**Keywords:** Gastrointestinal cancer, Medical research, Health care, Patient education, Public health, Quality of life

## Abstract

Health-related quality of life (HRQoL) has recently gained importance as treatment options for tumors of the upper GI tract lead to improved long-term survival. HRQoL is often estimated by physicians even though their reliability and the impact of outside factors such as contact time and level of medical education is unclear. Therefore, in this study we investigated the correlation between physicians’, students’, and patients’ assessment of HRQoL. 54 patients presenting with tumors of the upper GI tract were included and asked to fill out the standardized HRQoL questionnaires EORTC QLQ-C30 and QLQ-OG25. Attending physicians and medical students filled out the same questionnaires through estimation of patients’ HRQoL. Correlation was assessed through Pearson’s and Kendall’s τb coefficients. Physicians’ and patients’ assessments correlated for one out of six of the functional and a third of the symptom scores. Students’ and patients’ assessments correlated for one third of the functional and two thirds of the symptom scores. Students tended to underestimate patients’ symptom burden while physicians tended to overestimate it. Physicians failed to correctly assess several pathognomonic symptoms in this study. Students showed higher correlation with patients’ symptoms than physicians. Even so, this adds to mounting evidence that shows the benefit of using patient-reported outcomes as a gold standard regarding HRQoL.

## Introduction

Health-related quality of life (HRQoL) is gaining importance in daily practice as an outcome parameter for therapy of malignant and non-malignant diseases. Historically, tumors of the upper gastrointestinal (GI) tract ensue heavy reduction of HRQoL due to the tumor itself, the restricted food intake and the aggressive therapeutic approach^1,2^. Recently, developments in the therapeutic approach including neoadjuvant chemotherapy have led to improved long-term survival^3–7^. Consequently, attention has been directed on patients’ HRQoL during and after therapy, as HRQoL is known to decrease significantly after surgery^8,9^.

Ideally, HRQoL as an outcome parameter is obtained through direct questioning of the patient as patient reported outcome. Patient reported outcomes are defined as ‘any outcome evaluated directly by the patient himself and based on patient’s perception of a disease and its treatment(s)’ as per the definition published by the European Medicines Agency^10^. Various validated measures for assessment of patient reported HRQoL are available, most prominently in the form of questionnaires supplied by the European Organisation for Research and Treatment of Cancer.

Despite this, discussion of cancer-specific symptoms (CSS) and HRQoL is oftentimes not implemented in clinical practice and HRQoL sometimes is assessed by the physician based on the clinical impression due to time constraints or a patient’s inability to participate in standardized HRQoL measurements^11–13^. This clinician-reported HRQoL might then affect the pre- and postoperative access to additional treatment such as pain medication, physiotherapy or dietician advice^14^, potentially leading to over-as well as undertreatment.

Studies have demonstrated poor concordance of by-proxy and patient-reported assessments of quality of life in other patient cohorts^15–22^. This also applies to the postoperative phase. While patients understand the juristic need for technical information prior to surgery, they also wish for more information about the postoperative routine and HRQoL, which is not yet recognized by physicians^18^. Furthermore, HRQoL, especially social function, has been shown to be a prognostic factor for patient survival in various cancers, which underlines the interest in patients’ HRQoL^17,19^. Despite this scientific base, this method of patient-reported outcome (PRO) has not yet become the standard in all aspects of clinical care and systematic assessment of HRQoL is not performed comprehensively. There have been various studiess^15–22^, but so far there have been no studies on the congruence of the assessment of HRQoL made by physicians and patients for patients with tumors of the upper GI tract. Furthermore, though medical students often take part in patient assessment, up to now no studies have investigated the accuracy of students’ assessments of patients’ HRQoL.

For these reasons, we deemed it necessary to further investigate the differences in HRQoL evaluations by patients and physicians as well as medical students for tumors of the upper GI tract and evaluate the distribution of potential blind spots in by-proxy assessment of HRQoL by physicians or students in clinical practice.

## Methods

### Trial design and study population

Between August 2020 and September 2021, patients who presented at the outpatient clinic of RWTH Aachen University Hospital’s Department of Visceral Surgery with tumors of the upper GI tract were asked to participate in this study as part of the establishment of a data base for research on the relationship between HRQoL, nutrition and sarcopenia in patients with tumors of the upper GI tract^23^.

Patients who were under 18 years of age and those with insufficient level of German language were excluded from the study. Patients provided written, informed consent for participation in the study. This study was approved by the ethics committee of RWTH Aachen University (#EK 419/20). Two female medical students (with experience of at least three years of medical studies) and two physicians (one male, one female, with clinical experience of six or more years) took part in the completion of questionnaires as part of their work in the upper GI research group. All participating physicians and students were familiar with the applied questionnaires.

### Data collection

HRQoL was assessed as patients presented for staging appointments prior to or after neoadjuvant treatment and 6 months after surgery. Information was collected prior to surgery and then sorted into groups depending on their treatment status to account for the different situations of patients in various treatment phases. The t1 group is comprised of questionnaires by patients that had not yet received neoadjuvant therapy whereas patients that had completed neoadjuvant therapy were put in the t2 group. All patients were invited back for a follow-up appointment 6 months after receiving surgery and were assessed for HRQoL. The risk for learning bias was rated as low as there was a considerable amount of time between the assessments. Patients that presented in the outpatient clinic were asked to fill out a printed German version of the EORTC’s QLC-C30 and QLQ-OG25^15,16^ questionnaires after patient-physician contact. The QLC-C30 questionnaire consists of 30 questions about general cancer related functions and symptoms while the QLC-OG25 includes 25 questions that are specific to symptoms of upper GI tumors. For each question except two that assess overall health status and general quality of life (QoL) patients were asked to answer on a scale of 1 to 4 according to how much they agree with the statement or question, ranging from “not at all” to “very much”. For the two questions which assess overall health status and general QoL, patients are given options from one to seven, one being extremely low and seven being extremely high general QoL and health status.

As is common in German teaching hospitals, medical students were sent to the patient first to take a comprehensive patient history and inquire about weight loss, tumor type, prior systemic therapy and comorbidities without the presence of a physician.

Physicians then proceeded with their scheduled appointment with the patients. During the appointment, physicians had access to information collected by the medical students and asked further questions relevant to surgical plans, i.e. physical function, weight loss and nutrition concerns. After patient-physician contact, physicians and medical students were asked to fill out the same questionnaires as patients without having access to their answers. All participants, patients and medical personnel alike, used a pen and paper version of the questionnaires. Participating physicians were aware of the content and questions of the questionnaires and not artificially restricted in their interaction with the patient.

### Statistical analysis

The HRQoL questionnaires were processed according to the official EORTC scoring manuals. Answers were adapted to a scale of one to a hundred using linear transformation and then combined into six functional and nine symptom scales for the QLQ-C30 questionnaire as well as sixteen symptom scales for the QLQ-OG25 questionnaire. Similar to a previous study^24^, we chose to not use all scales, instead limiting the analysis to the six QLQ-C30 functional scores and the six QLQ-OG25 multiple-item scales. Answers were grouped into “prior to neoadjuvant therapy” (from hereon t_1_), “post neoadjuvant therapy” (from hereon t_2_), and “6 months post operation” (from hereon t_3_). For each subgroup, physicians’ and students’ answers were compared to patients’ answers using Spearman rho correlation coefficient. To adjust for multiple testing, we conducted Bonferroni correction, resulting in an adjusted significance level of α = 0.008.

## Results

A total of n = 54 patients were included in this study. Tumor localisation was in the esophagogastric junction in 42.6% of cases and in the esophagus or stomach in the remaining patients. Most patients were male (79.6%) and up to 70 years of age (61.1%). For an overview of general information on the study cohort see Table 1.

**Table 1:** General cohort information on the study cohort.

		n	relative in %
*Total patient cohort*		54	100
Tumor localisation	Esophagogastric junction	23	42.6
	Esophageal	15	27.8
	Gastric	16	29.6
ASA score	ASA 2	18	33.3
	ASA 3	28	51.9
	ASA 4	4	7.4
	Missing	4	7.4
Sex	Female	11	20.4
	Male	43	79.6
Age	< = 70 years	33	61.1
	> 70 years	21	38.9
MUST	0	22	40.7
	1	13	24.1
	≥ 2	18	33.3
	Missing	1	1.9
History of smoking in the previous 10 years	Smoker	19	35.2
	Non smoker	34	63.0
	Missing	1	1.9
COPD	COPD	2	3.7
	No COPD	51	94.4
	Missing	1	1.9
Hypertension	Hypertension	33	61.1
	No hypertension	20	37.0
	Missing	1	1.9
Coronary heart disease	CHD	9	16.7
	No CHD	44	81.5
	Missing	1	1.9

Values are indicated as n (%).

*ASA* American Society of Anesthesiologists; *MUST* Malnutrition Universal Screening Tool; *COPD* chronic obstructive pulmonary disease; *CHD* coronary heart disease.

The t_1_ group was comprised of 22 pairs of physician–patient completed questionnaires and 23 pairs of student-patient completed questionnaires. Data for one physician completed questionnaire was missing. The t_2_ group was made up of 24 complete data sets of physician, student and patient completed questionnaires. 13 patients returned for a follow up 6 months after their surgery, 7 of those patients had already participated in HRQoL assessment prior to their surgery.

### Comparison of physicians and patients

Physicians showed significant correlation with patients in their assessment for only two out of six functional scales (physical functioning and cognitive functioning). Doctors significantly correlated with patients in their assessment of physical functioning at all assessed time points during the study (*p*-value t_1_ < 0.001/t_2_ = 0.037/t_3_ = 0.006). Physicians and patients also correlated in their assessment of cognitive functioning (*p*-value t_2_ = 0.019), a score students failed to show correlation for.

After Bonferroni correction, a significant correlation only remained for physical functioning in the t_1_ and t_3_ groups.

In contrast to the students, doctors showed significant correlation with patients in only 3 of the 6 symptom scores. Physicians and patients showed highly significant correlation for odynophagia (*p*-value t_1_ = 0.005) prior to neoadjuvant therapy. After neoadjuvant therapy however, a correlation for odynophagia could not be shown.

After adjustment for Type I error inflation, the only correlation that remained significant were the above mentioned correlation for odynophagia as well as the assessment of reflux 6 months after surgery (*p* = 0.006).

Concerning the different assessment groups, adjusted significant correlations between physicians and patients were shown for 2 out of 12 (16.67%) scores in the t_1_ group, 0 out of 12 in the t_2_ group and 2 out of 12 (16.67%) in the t_3_ group.

**Figure 1:**
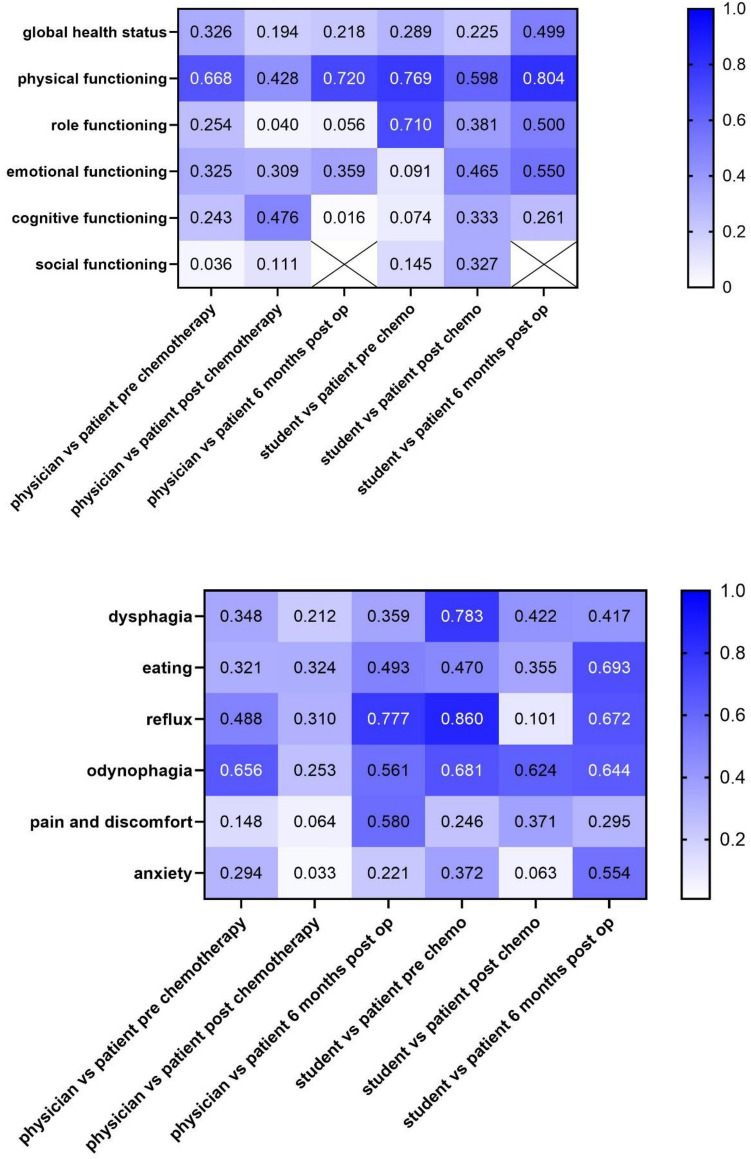
Overview of correlation coefficients for the EORTC’s QLQ-C30 (**a**) and QLQ-OG25 (**b**), darker color indicates higher correlation coefficients. *EORTC* European Organisation for Research and Treatment of Cancerspaces marked with an X are indicative of cases where calculation of correlation coefficients was not possible.

**Figure 2:**
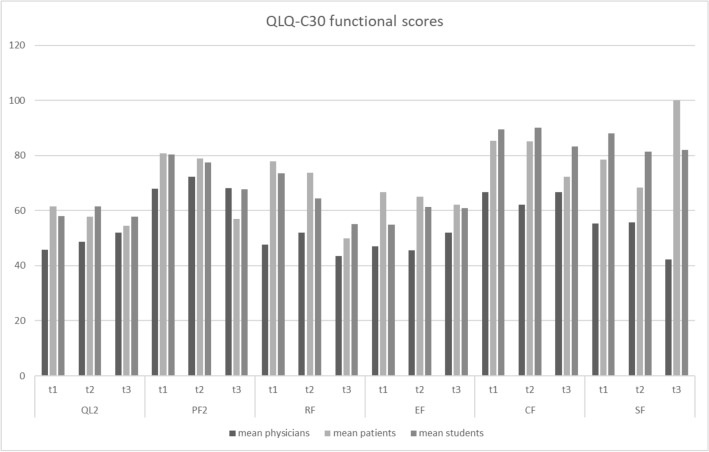
Assessment of quality of life (QoL) and functioning scales by physicians, students and patients. *QL2* QoL, *PF2* physical functioning, *RF2* role functioning, *EF* emotional functioning, *CF* cognitive functioning, *SF* social functioning.

**Figure 3:**
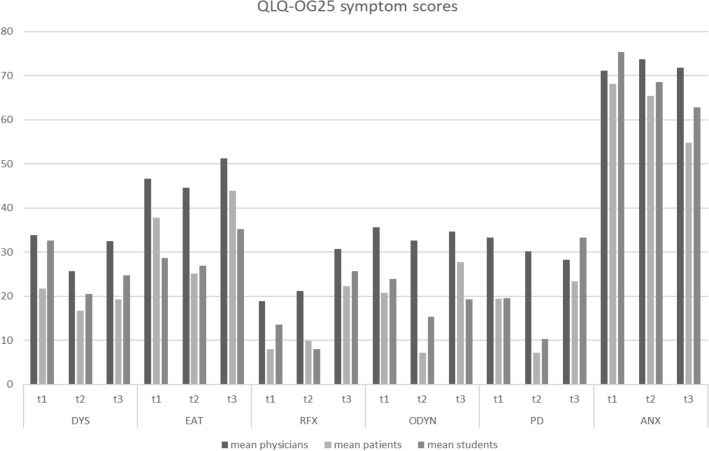
Assessment of selected symptoms scores by physicians, students and patients. *DYS* dysphagia, *EAT* eating, *RFX* reflux, *ODYN* odynophagia, *PD* pain and discomfort, *ANX* anxiety.

For an overview of all correlation coefficients see Fig. 1. Graphical illustrations of the differences in functioning scales and symptom scores can be seen in Figs. 2 and 3.

### Comparison of medical students and patients

When comparing the assessments of patients and medical students, significant correlations could be found for three out of the six functional scales. Most notably and consistent with physicians’ assessment, students’ assessment of physical functioning correlated significantly with patients’ indications at all assessed points during the treatment (*p*: t_1_ < 0.001/t_2_ = 0.002/t_3_ < 0.001). Significant correlation regarding role functioning was shown in the t_1_ group (*p*-value t_1_ < 0.001) and assessment of emotional functioning was shown to significantly correlate in the t_2_ group (*p* = 0.022). For correlation coefficients see Fig. 1.

After adjusting for multiple testing, the assessment of emotional functioning did no longer show significant correlation in the t_2_ group.

Regarding the QLQ-OG25 symptom scores, students and patients showed significant correlation at some point for 4 of the six scores (dysphagia, eating, reflux, odynophagia). After Bonferroni correction, a significant correlation could still be shown for dysphagia, eating and reflux in the t_1_ group (dysphagia: *p*-value t_1_ < 0.001, eating: *p*-value t_1_ < 0.001, reflux: *p*-value t_1_ = 0.001).

An adjusted significant correlation could also be shown for all groups for the symptom odynophagia (*p*-value t_1_ = 0.006/t_2_ < 0.001/t_3_ < 0.001 ).Concerning the different assessment groups, adjusted significant correlations between students and patients were shown for 6 out of 12 scores (50%) in the t_1_ group, 3 out of 12 scores (25%) in the t_2_ group and 4 out of 12 scores (33.33%) in the t_3_ follow-up group.

## Discussion

Patients and physicians might have diverging views on therapy success, side effects and illness burden. Ultimately decisive are the patients’ perception of symptoms and health restrictions. However, physicians sometimes might make estimations of patients’ health for therapy decisions^25^. In line with the findings of Kong et al. and others, we found the HRQoL of the participants of this study to be heterogenous and it seemed to be almost comparable to the general population 6 months after surgery^8,26,27^. This is important for treating physicians, as patients might be affected differently by the symptoms of the cancer or its therapy in their HRQoL. Studies have shown both under-as well as overestimation of certain symptoms and aspects of HRQoL by physicians^25,28–30^. Furthermore, Celli et al. and Tamir et al. have shown that a significant difference in HRQoL assessments can be observed for patients with COPD and diabetes between patients and physicians^29,30^. Another important finding in these studies is, that while about 90% of patients admit not being honest with their physicians during appointments, most physicians were aware of this and did not try to overcome this gap^30^. This might indicate a certain blindness for patients’ actual clinical problems. In another large multicenter study, most patients reported deficient discussing and care for cancer-specific symptoms^13^.

Medical students differ from physicians in that they are not involved in treatment choices. They might therefore make unbiased estimations of patients’ HRQoL. We hypothesized that students might therefore be more concise in estimation of patients’ HRQoL.

While in our study students’ perception of HRQoL showed a significant correlation after Bonferroni correction with patients in their assessments for 33.33% of functional scales and 66.67% of analysed symptom scales, physicians did so for only a sixth of the functional scales and a third of symptom scales. This confirms our hypothesis that students might be more concise at assessing their patients’ QoL than physicians. Notably, doctors did not correlate with patients for the scores dysphagia and eating, both of which are highly relevant to the course of treatment and usually assessed during the initial appointment. This mirrors the findings of Rammant et al. who found that for patients that received treatment for prostate cancer, physicians were likely to underreport urinary toxicity, which is not only frequent after treatment but has also been shown to correlate with HRQoL^28^.

It should be noted that due to the clinical setting, students spent a considerably longer amount of time with the patient. The added time students have with the patient can be used to take a more thorough patient history and build a deeper rapport with patients. Even so, Tamir et al. showed that familiarity was not a factor that could explain the difference in HRQoL assessment for outpatient patients with diabetes, which puts time restrictions into the background as a confounder in our study setting.

Looking at the results of our study, physicians tend to underestimate the HRQoL and functionality of their patients whereas students tend to overestimate these same aspects. This suggests that either students and physicians obtain different information from their patients or that there is a considerable difference in the way they process the same information. As Celli et al. have shown, about 90% of patients are dishonest with their physicians^30^. Furthermore, downplaying symptoms is common in elder patients^31–35^ and as such may lead to the slight overestimation of HRQoL and underestimation of symptom burden that could be observed in the assessments of medical students.

Physicians on the other hand have had considerably more encounters with patients and may tend to view their statements in context with other patients they have previously seen, including patients with prolonged postoperative courses. Furthermore, it seems reasonable that the bad prognosis and overall survival for upper GI cancers might cause a cognitive or availability bias in more experienced surgeons which has otherwise been shown to be linked with diagnostic errors^36^.

Sibeoni et al. conducted a qualitative study based on semi-structured interviews to assess the influencing factors on patients’ HRQoL while undergoing cancer therapy and highlight the relationship with the physician as being positively associated with HRQoL^37^. This underlines the importance of a close relationship and honest communication between patient and treating physician.

Even though, contrary to an underestimation of symptom burden, overestimation of the patients’ symptoms is unlikely to restrict access to therapy in a standard surgical setting, it might do so in other settings, i.e., palliative or conservative approaches, if the patient is falsely deemed unfit for interventions due to perceived bad overall health or resilience.

The results of this study must be regarded with some limitations. Firstly, only a small number of patients was included, owing to the low incidence of upper GI tumors in Germany, which might prevent application of these results to the general patient cohort. It should be further noted that this study was conducted under COVID-19 caused restrictions, therefore questions about leisure activities might be less indicative of tumor-caused than COVID-19-related restrictions as remarked by patients.

Furthermore, only two physicians and two students participated in the assessments. Even though they reflected some of the variety through their different stages of training, it is not certain that they accurately reflect the average physician and as such, a more thorough study with a broader range of physicians is required.

Nonetheless, our findings represent the average difference in QoL assessment that can be expected in a routine clinical setting. Patients’ perception of QoL is the absolute gold standard and should by no means be questioned. However, as by proxy estimation of HRQoL is far from uncommon in clinical settings, this study conveys important information on the inaccuracy of symptom estimation that every oncologically practicing physician should keep in mind. Notwithstanding the aforementioned limitations, this is to our knowledge the first study to assess the difference in physicians’ and students’ perception of oncological patients’ HRQoL. The results are remarkable and justify further clinical studies, ideally in a multicenter setting.

## Conclusions

Both medical students and attending surgeons show highly significant correlations in their assessment of patients’ HRQoL when compared with the patients themselves. However, students tended to fare better in estimating their patients’ HRQoL than physicians in this study. Physicians showed lower correlation coefficients than students for almost all examined functional and especially symptom scales and tended to overestimate symptom burden while simultaneously underestimating HRQoL. This could be attributed to time pressure and clinical routine as well as a physician bias caused by the poor prognosis of cancers of the upper GI tract. Further elaborate studies are needed to correctly assess singular influencing factors on the assessment of HRQoL in patients with upper GI tumors as well as other tumors for different treatment stages.

In any case, HRQoL should be obtained through patient questionnaires not only in clinical studies but also as a routine part of surgical preparation as it provides the physician with a holistic view of the patient.

## Data Availability

Data is available upon reasonable request from the corresponding author.

## References

[1] Fuchs H, Holscher AH, Leers J (2016). Long-term quality of life after surgery for adenocarcinoma of the esophagogastric junction: Extended gastrectomy or transthoracic esophagectomy?. Gastric Cancer.

[2] Talagala IA, Arambepola C (2018). Changes in quality of life following initial treatment of oesophageal carcinoma: A cohort study from Sri Lanka. BMC Cancer.

[3] Mittal SK, Abdo J, Adrien MP, Bayu BA, Kline JR, Sullivan MM, Agrawal DK (2021). Current state of prognostication, therapy and prospective innovations for Barrett’s-related esophageal adenocarcinoma: A literature review. J. Gastrointest. Oncol..

[4] Gotze TO, Piso P, Lorenzen S (2021). Preventive HIPEC in combination with perioperative FLOT versus FLOT alone for resectable diffuse type gastric and gastroesophageal junction type II/III adenocarcinoma-the phase III “PREVENT”-(FLOT9) trial of the AIO/CAOGI/ACO. BMC Cancer.

[5] Chowdappa R, Dharanikota A, Arjunan R, Althaf S, Premalata CS, Ranganath N (2021). Operative outcomes of minimally invasive esophagectomy versus open esophagectomy for resectable esophageal cancer. South Asian J. Cancer..

[6] Zhou YJ, Lu XF, Meng JL (2021). Neo-adjuvant radiation therapy provides a survival advantage in T3–T4 nodal positive gastric and gastroesophageal junction adenocarcinoma: A SEER database analysis. BMC Cancer.

[7] Arnold, M. R. M., Lam, F., Bray, F., Ervik, M. & Soerjomataram I. ICBP SURVMARK-2 online tool: International cancer survival benchmarking. Lyon, France: International Agency for Research on Cancer. Available from: http://gco.iarc.fr/survival/survmark, accessed [31/01/2022]. (2019).

[8] Hu Y, Vos EL, Baser RE, Schattner MA, Nishimura M, Coit DG, Strong VE (2021). Longitudinal analysis of quality-of-life recovery after gastrectomy for cancer. Ann. Surg. Oncol..

[9] Barbour AP, Lagergren P, Hughes R, Alderson D, Barham CP, Blazeby JM (2008). Health-related quality of life among patients with adenocarcinoma of the gastro-oesophageal junction treated by gastrectomy or oesophagectomy. Br. J. Surg..

[10] EMA. Reflection paper on the use of patient reported outcome (PRO) measures in oncology studies [Draft]. European Medicines Agency, Oncology Working Party; Doc. Ref. EMA/CHMP/292464/2014. (2014).

[11] Basch E (2017). Patient-reported outcomes-harnessing patients' voices to improve clinical care. N Engl. J. Med..

[12] Laugsand EA, Sprangers MA, Bjordal K, Skorpen F, Kaasa S, Klepstad P (2010). Health care providers underestimate symptom intensities of cancer patients: a multicenter European study. Health Qual. Life Outcomes..

[13] Smith TG, Troeschel AN, Castro KM (2019). Perceptions of patients with breast and colon cancer of the management of cancer-related pain, fatigue, and emotional distress in community oncology. J. Clin. Oncol..

[14] Cleeland CS, Gonin R, Hatfield AK, Edmonson JH, Blum RH, Stewart JA, Pandya KJ (1994). Pain and its treatment in outpatients with metastatic cancer. N Engl. J. Med..

[15] Lagergren P, Fayers P, Conroy T (2007). Clinical and psychometric validation of a questionnaire module, the EORTC QLQ-OG25, to assess health-related quality of life in patients with cancer of the oesophagus, the oesophago-gastric junction and the stomach. Eur. J. Cancer..

[16] Aaronson NKAS, Bergman B, Bullinger M, Cull A, Duez NJ, Filiberti A, Flechtner HFS, de Haes JCJM, Kaasa S, Klee MC, Osoba D, Razavi D, Rofe PBSS, Sneeuw KCA, Sullivan M, Takeda F (1993). The European organisation for research and treatment of cancer QLQ-C30: A quality-of-life instrument for use in international clinical trials in oncology. J. Natl. Cancer Inst..

[17] Husson O, de Rooij BH, Kieffer J (2020). The EORTC QLQ-C30 summary score as prognostic factor for survival of patients with cancer in the “Real-World”: Results from the population-based PROFILES registry. Oncologist..

[18] McNair AGK, MacKichan F, Donovan JL (2016). What surgeons tell patients and what patients want to know before major cancer surgery: A qualitative study. BMC Cancer..

[19] van Kleef JJ, Dijksterhuis WPM, van den Boorn HG (2021). Prognostic value of patient-reported quality of life for survival in oesophagogastric cancer: Analysis from the population-based POCOP study. Gastric Cancer.

[20] Janse AJ, Gemke RJ, Uiterwaal CS, van der Tweel I, Kimpen JL, Sinnema G (2004). Quality of life: Patients and doctors don’t always agree: A meta-analysis. J. Clin. Epidemiol..

[21] Atkinson TM, Rogak LJ, Heon N (2017). Exploring differences in adverse symptom event grading thresholds between clinicians and patients in the clinical trial setting. J. Cancer Res. Clin. Oncol..

[22] Bergerot CD, Philip EJ, Bergerot PG (2021). Discrepancies between genitourinary cancer patients’ and clinicians' characterization of the Eastern cooperative oncology group performance status. Cancer..

[23] Oberhoff G, Schooren L, Vondran F (2024). Impairment of nutritional status and quality of life following minimal-invasive esophagectomy-a prospective cohort analysis. Cancers (Basel)..

[24] Rogers SN, Waylen AE, Thomas S (2020). Quality of life, cognitive, physical and emotional function at diagnosis predicts head and neck cancer survival: Analysis of cases from the head and neck 5000 study. Eur. Arch. Otorhinolaryngol..

[25] Xiao C, Polomano R, Bruner DW (2013). Comparison between patient-reported and clinician-observed symptoms in oncology. Cancer Nurs..

[26] Kobayashi D, Kodera Y, Fujiwara M, Koike M, Nakayama G, Nakao A (2011). Assessment of quality of life after gastrectomy using EORTC QLQ-C30 and STO22. World J. Surg..

[27] Kong H, Kwon OK, Yu W (2012). Changes of quality of life after gastric cancer surgery. J. Gastric Cancer..

[28] Rammant, E., Ost, P., Swimberghe, M. & et al. Patient-versus physician-reported outcomes in prostate cancer patients receiving hypofractionated radiotherapy within a randomized controlled trial. *Strahlenther. Onkol*. **195**(5), 393–401 (2019). Patienten- versus arztberichtete Ergebnisse von Prostatakrebspatienten nach hypofraktionierter Radiotherapie innerhalb einer randomisierten kontrollierten Studie. 10.1007/s00066-018-1395-y 10.1007/s00066-018-1395-y 30406289

[29] Tamir O, De-Paz NS, Dvir D, Heymann AD (2018). Comparing assessment of diabetes-related quality of life between patients and their physicians. Health Qual. Life Outcomes..

[30] Celli B, Blasi F, Gaga M (2017). Perception of symptoms and quality of life-comparison of patients’ and physicians’ views in the COPD MIRROR study. Int. J. Chron. Obstruct. Pulmon. Dis..

[31] Rubio R, Palacios B, Varela L (2021). Quality of life and disease experience in patients with heart failure with reduced ejection fraction in Spain: A mixed-methods study. BMJ Open..

[32] Ganzer H, Rothpletz-Puglia P, Byham-Gray L, Murphy BA, Touger-Decker R (2015). The eating experience in long-term survivors of head and neck cancer: A mixed-methods study. Support Care Cancer..

[33] Svensson S, Linell P, Kjellgren KI (2008). Making sense of blood pressure values in follow-up appointments for hypertension. Int. J. Cardiol..

[34] Isaksson RM, Brulin C, Eliasson M, Naslund U, Zingmark K (2013). Older women's prehospital experiences of their first myocardial infarction. J. Cardiovasc. Nurs..

[35] Kohler, K., Regner, A., Koenigsmann, M., Franke, A. & Frommer, J. [Illness perceptions of patients suffering from acute leukaemia one week after diagnosis]. *Z. Psychosom. Med. Psychother*. **51**(4), 388–402 (2005). Subjektive Krankheitsvorstellungen bei Patienten mit akuter Leukamie eine Woche nach Diagnostellung. 10.13109/zptm.2005.51.4.388 10.13109/zptm.2005.51.4.388 16402336

[36] Watari T, Tokuda Y, Amano Y, Onigata K, Kanda H (2022). Cognitive bias and diagnostic errors among physicians in Japan: A self-reflection survey. Int. J. Environ. Res. Public Health..

[37] Sibeoni J, Picard C, Orri M, Labey M, Bousquet G, Verneuil L, Revah-Levy A (2018). Patients’ quality of life during active cancer treatment: A qualitative study. BMC Cancer..

